# Comparative immunophenotyping of peripheral blood lymphocyte subsets in pulmonary tuberculosis and nontuberculous mycobacterial pulmonary disease: a retrospective study

**DOI:** 10.3389/fimmu.2025.1682099

**Published:** 2025-10-15

**Authors:** Shuai Ren, Ruifang Chen, Ping Xu, Zhangyan Zhao, Haicheng Tang

**Affiliations:** Department of Pulmonology, Shanghai Public Health Clinical Center, Shanghai, China

**Keywords:** pulmonary tuberculosis, nontuberculous mycobacteria, differential diagnosis, lymphocyte subsets, flow cytometry, biomarkers

## Abstract

**Objective:**

To compare peripheral blood lymphocyte profiles between pulmonary tuberculosis (PTB) and nontuberculous mycobacterial pulmonary disease (NTMPD) patients and explore whether these immunophenotypic differences may assist future diagnostic algorithms.

**Methods:**

This retrospective study analyzed clinical data of 78 PTB patients, 73 NTMPD patients, and 80 healthy controls from the Shanghai Public Health Clinical Center between February 2024 and February 2025. Peripheral blood lymphocyte subsets were measured using flow cytometry. Logistic regression and receiver operating characteristic (ROC) curve analyses were performed to identify and assess diagnostic markers.

**Results:**

No significant differences in age or body mass index (BMI) were found among the three groups (P > 0.05), but gender distribution differed significantly (P < 0.05). PTB patients had a higher proportion of diabetes mellitus (P < 0.05), while NTMPD patients had a higher prevalence of structural lung disease (P < 0.05). PTB patients also showed higher platelet counts and CD3^+^, CD4^+^, and CD45+ lymphocyte counts (gated within the CD45+SSC-Alow population) compared to NTMPD patients (all P < 0.05). ROC analysis indicated that CD4^+^ lymphocytes had an area under the curve (AUC) of 0.722, with a sensitivity of 65.9% and specificity of 76.7%.

**Conclusion:**

Significant differences in peripheral blood lymphocyte subsets exist between PTB and NTMPD patients. The CD4^+^ counts differed significantly between the two diseases, suggesting that immune profiling could contribute to a broader diagnostic framework once prospectively validated.

## 1 Introduction

Tuberculosis (TB) is a chronic infectious disease caused by the Mycobacterium tuberculosis complex (MTBC) (1). Nontuberculous mycobacteria (NTM) refer to mycobacteria other than MTBC and Mycobacterium leprae, and over 190 species have been identified (2). Despite their taxonomic similarities, TB and NTM diseases differ significantly in pathogenesis, treatment, and outcomes. TB remains a major global public health challenge. In 2022, it was reported that there were over 10 million new TB cases worldwide, with approximately 748,000 new cases in China, accounting for 7.1% of the global total (3). Meanwhile, the incidence of NTM diseases is increasing globally (4). Although China lacks national - scale epidemiological data, studies indicate that the proportion of NTM in clinical isolates rose significantly from 4.3% in 1979 to 22.9% in 2010 (5).

However, in clinical practice, accurately differentiating pulmonary tuberculosis (PTB) from nontuberculous mycobacterial pulmonary disease (NTMPD) remains challenging. First, the imaging features of the two conditions on chest computed tomography (CT) often overlap (6). Second, traditional mycobacterial culture methods are time-consuming (taking weeks), which fails to meet the need for rapid differential diagnosis (7). Although molecular diagnostic techniques such as metagenomic next-generation sequencing (mNGS) can provide pathogenetic evidence, they are costly and not widely available in primary healthcare settings (8). Early differentiation is critical because empiric anti-tuberculosis therapy may be initiated in hospital; inappropriate treatment for NTMPD can delay effective therapy (macrolides/rifamycins), increase drug toxicity, and promote antimicrobial resistance. Therefore, characterizing disease-specific immune signatures that might complement conventional diagnostics in future multicenter studies is warranted.

Peripheral blood lymphocyte subsets, key indicators of immune status, have shown potential in diagnosing and differentiating infectious diseases. This study aims to compare the clinical characteristics and peripheral blood lymphocyte subset distributions among PTB patients, NTMPD patients, and healthy controls. It explores the potential value of specific lymphocyte subsets in differentiating PTB from NTMPD, aiming to provide immune-profile data that may inform future diagnostic strategies.

## 2 Data and methods

### 2.1 General information

#### 2.1.1 Study subjects

The study enrolled 78 PTB patients (50 male, 28 female, mean age 68.7 ± 14.15 years), 73 NTMPD patients (29 male, 44 female, mean age 71.67 ± 9.99 years), and 80 healthy controls (40 male, 40 female, mean age 70.86 ± 12.56 years) from the Shanghai Public Health Clinical Center between February 2024 and February 2025. Healthy controls were recruited from routine health-check visitors at the same center during the same period. They had no history of TB or NTMPD, normal chest X-ray, and negative interferon-γ release assay. Formal matching by age and sex was not performed *a priori*, but baseline characteristics were comparable across groups (**Table 1**). The hospital’s medical ethics committee approved the study (approval No. 2023-S010-02).

**Table 1:** Comparison of basic characteristics among the three groups.

Variable	PTB arm (n = 78)	NTMPD arm (n = 73)	Control arm (n = 80)	Statistical measure	*P*- value
Female n (%)	50 (64.1)	29 (39.72)	40 (50)	-2.45	0.014
Age mean ± SD	68.7 ± 14.15	71.67 ± 9.99	70.86 ± 12.56	-0.727	0.467
BMI	20.76 (18.37-23.94)	20.31 (18.37-22.50)	20.24 (18.55-23.02)	-0.811	0.417
Hypertension n (%)	32 (41.02)^a^	32 (43.84)^a^	0	-0.096	0.759
Diabetes n (%)	19 (24.35)^a^	2 (2.73)^a^	0	-3.049	0.002
Structural lung disease n (%)	5 (6.41)^a^	27 (36.98)^a^	0	-3.411	0.000

BMI = weight (kg)/height (m)²; ^a^ *P* < 0.05 vs. healthy control group; statistical values represent comparison between PTB and NTMPD groups; *P* value refers to comparison between PTB and NTMPD groups.

#### 2.1.2 Inclusion and exclusion criteria

Inclusion Criteria: (1) PTB diagnosis met the confirmation criteria in the “2018 Pulmonary Tuberculosis Primary Care Guidelines”; (2) NTMPD diagnosis complied with the confirmation criteria in the “2020 Guidelines for Diagnosis and Treatment of Nontuberculous Mycobacterial Diseases”.

Exclusion Criteria: (1) Patients aged under 18; (2) Patients with malignant tumors, immunosystem diseases, or severe hepatic/renal dysfunction; (3) Patients not newly diagnosed with PTB or NTMPD; (4) Patients with both PTB and NTMPD; (5) Patients who used immunosuppressants within the last 3 months; (6) Patients who received antituberculosis treatment; (7) Patients with incomplete clinical data.

### 2.2 Research methods

Baseline demographic data (age, sex, height, weight, smoking history) and comorbidities (hypertension, diabetes, structural lung disease) were recorded on admission, and BMI was calculated.

Fasting venous blood (5 mL) was collected on the morning of admission before any anti-mycobacterial or antimicrobial therapy was initiated; samples for complete blood count, CRP and PCT were obtained simultaneously.

Lymphocyte subset quantification:Peripheral blood (50 μL) was stained with BD Multitest™ TBNK 6-color antibody cocktail (CD3-FITC, CD16-PE/CD56-PE, CD45-PerCP-Cy5.5, CD4-PE-Cy7, CD19-APC, CD8-APC-Cy7) in BD Trucount™ absolute-count tubes following the manufacturer’s protocol (BD Biosciences). After 15 min incubation at room temperature in the dark, 450 μL of 1× BD FACS lysing solution was added and incubated for another 15 min. Samples were acquired on a BD FACSCanto II within 2 h. Absolute counts (cells/μL) were calculated using the built-in BD Trucount™ bead formula. All tests were completed within 4 h of blood collection and monitored daily with BD Multi-Check control beads according to our CAP-accredited laboratory SOP (SM351).

Gating sequence: Lymphocytes were identified on CD45 vs SSC-A, doublets were excluded using FSC-H vs FSC-A, and T cells (CD3+), helper T cells (CD3+CD4+), cytotoxic T cells (CD3+CD8+), B cells (CD19+), and NK cells (CD3−CD16+CD56+) were enumerated within the CD45+ lymphocyte subset (CD45+SSC-Alow), excluding myeloid and other non-lymphoid cells.

Reproducibility note: The original list-mode (.fcs) files were automatically purged by the hospital’s data-retention policy; therefore, a graphical gating scheme could not be generated, but the narrative sequence above ensures full reproducibility.

### 2.3 Statistical methods

All statistical analyses were performed using SPSS 27.0 (IBM Analytics) software. Normally distributed continuous data were expressed as mean ± standard deviation, non-normally distributed continuous data as median (interquartile range), and categorical data as frequency (percentage). The t-test was used for normally distributed data, the rank-sum test for non-normally distributed data, and the chi-square test for categorical data. Univariate and multivariate binary logistic regression analyses were performed on lymphocyte subsets, followed by ROC analysis. Multicollinearity was assessed by a variance inflation factor (VIF) < 5. Odds ratios (OR) and 95% confidence intervals (CI) were calculated.

## 3 Results

### 3.1 Comparison of clinical data

This study included 78 PTB patients, 73 NTMPD patients, and 80 healthy controls. No significant differences in age or BMI were found among the three groups (*P* > 0.05), ensuring comparability. However, significant gender distribution differences existed between the PTB and NTMPD groups (*P* < 0.05). Additionally, significant differences in hypertension, diabetes, and structural lung disease were observed among the three groups (*P* < 0.05) (**Table 1**).

### 3.2 Comparison of blood routine, CRP, and PCT

The PTB group had significantly higher platelet counts than the NTMPD group (P = 0.027), whereas differences versus controls were not significant (P > 0.05). Additionally, no significant differences were found between the PTB and NTMPD groups in red blood cell count, hemoglobin, neutrophils, lymphocytes, CRP, and PCT (*P* > 0.05). However, both the PTB and NTMPD groups showed significant differences in these blood parameters compared to the control group (*P* < 0.05) (**Table 2**).

**Table 2:** Comparison of blood routine, CRP, and PCT among the three groups.

Variable	PTB arm (n = 78)	NTMPD arm (n = 73)	Control arm (n = 80)	Statistical measure	*P*- value
RBCs	4.08 (3.65-4.53)^a^	4.04 (3.76-4.38)^a^	4.88 (4.4-5.07)	-0.212	0.832
Hb	120.5 (107.5-136.25)^a^	121 (111-130)^a^	140 (132-152.75)	-0.076	0.939
PLT	232.5 (184.75-321.25)	203 (165-232)^a^	220 (200-279)	-2.207	0.027
NEU	4.50 ± 2.06^a^	5.01 ± 3.19^a^	3.96 ± 1.36	-0.450	0.653
LYM	1.15 (0.86-1.60)^a^	0.97 (0.69-1.36)^a^	2.02 (1.9-2.03)	-1.766	0.077
CRP	22.59 ± 31.16^a^	31.38 ± 50.46^a^	3.02 ± 1.62	-0.101	0.919
PCT	0.09 ± 0.11^a^	0.07 ± 0.06^a^	0.02 ± 0.01	-1.122	0.262

RBCs, Red Blood Cells; Hb, Hemoglobin; PLT, Platelets; NEU, Neutrophils; lym, Lymphocytes; CRP, C-reactive protein; PCT, Procalcitonin; ^a^ *P* < 0.05 vs. healthy control group; statistical values represent comparison between PTB and NTMPD groups; P value refers to comparison between PTB and NTMPD groups.

### 3.3 Comparison of peripheral blood lymphocyte subsets

Significant differences were found in CD3, CD4, and CD45 levels between the PTB and NTMPD groups (*P* < 0.05), while CD8, CD19, and CD16CD56 levels showed no significant differences (*P* > 0.05). Compared to the control group, all these subsets (CD3, CD4, CD8, CD45, CD19, CD16CD56) in both the PTB and NTMPD groups exhibited significant differences (*P* < 0.05) (**Table 3**). Within the PTB group, CD4^+^ counts did not differ significantly between diabetic and non-diabetic patients (*P* = 0.881, **Supplementary Table S1**), suggesting that diabetes status had minimal influence on the main findings. Distribution summaries are provided in **Supplementary Figure S2**.

**Table 3:** Comparison of peripheral blood lymphocyte subsets among the three groups.

Variable	PTB arm (n = 78)	NTMPD arm (n = 73)	Control arm (n = 80)	Statistical measure	*P*- value
CD3	810.64 (546.72-1034.93)^a^	605.15 (446.59-803.80)^a^	1258.98 (932.58-1506.44)	-2.411	0.016
CD8	287.12 ± 183.01^a^	259.69 ± 147.79^a^	697.05 ± 147.57	-0.391	0.696
CD4	520.46 (347.50-651.39)^a^	331.35 (232.57-424.49)^a^	989.85 (763.25-1302.75)	-3.566	0.000
CD45	1110.69 (852.02-1502.41)^a^	886.89 (697.55-1270.53)^a^	1763.74 (1438.61-2251.82)	-2.331	0.020
CD19	125.02 (67.45-182.86)^a^	96.57 (47.36-166.42)^a^	386.53 (295.73-460.21)	-1.601	0.109
CD16CD56	223.94 ± 114.88^a^	250.69 ± 195.79^a^	383.92 ± 228.53	-0.387	0.699

a P < 0.05 vs. healthy control group; statistical values represent comparison between PTB and NTMPD groups; P value refers to comparison between PTB and NTMPD groups. All values were acquired strictly within the CD45+ lymphocyte subset (CD45+SSC-Alow) using BD Trucount™ absolute-count tubes and are expressed as cells/μL.

### 3.4 Univariate logistic regression analysis of lymphocyte subsets

Univariate logistic regression analysis was used to identify differences between the PTB and NTMPD groups. CD3, CD4, and CD45 were found to be statistically significant (*P* < 0.05) (**Table 4**).

**Table 4:** Univariate logistic regression analysis of peripheral blood lymphocyte subsets between PTB and NTMPD groups.

Variable	Estimate	SE	WALD	*P*- value	OR (95%CI)
CD3	-0.002	0.001	6.532	0.011	0.998 (0.997-1.000)
CD8	-0.001	0.001	0.600	0.439	0.999 (0.996-1.002)
CD4	-0.004	0.001	11.205	0.000	0.996 (0.993-0.998)
CD45	-0.001	0.001	4.936	0.026	0.999 (0.998-1.000)
CD19	-0.006	0.003	3.614	0.057	0.994 (0.988-1.000)
CD16CD56	0.001	0.002	0.494	0.824	1.001 (0.998-1.004)

SE, Standard Error; QALD, Wald Test; OR, Odds Ratio.

### 3.5 Multivariate logistic regression analysis of lymphocyte subsets

CD3, CD4, CD45, and CD19 were selected as independent variables for multivariate logistic regression analysis. The results showed that CD4 (OR = 0.992, 95% CI: 0.984 - 1.000) was statistically significant in differentiating PTB from NTMPD (*P* < 0.05) (**Table 5**).

**Table 5:** Multivariate logistic regression analysis of peripheral blood lymphocyte subsets between PTB and NTMPD groups.

Variable	Estimate	SE	WALD	*P*- value	OR (95%CI)
CD3	0.001	0.003	0.139	0.709	1.001 (0.995-1.007)
CD4	-0.008	0.004	4.248	0.039	0.992 (0.984-1.000)
CD45	0.002	0.002	0.901	0.343	1.002 (0.998-1.005)
CD19	-0.006	0.004	2.111	0.146	0.994 (0.986-1.002)

SE, Standard Error; QALD, Wald Test; OR, Odds Ratio.

### 3.6 ROC curve of CD4^+^ lymphocyte counts for distinguishing PTB from NTMPD in the study cohort

**Figure 1:**
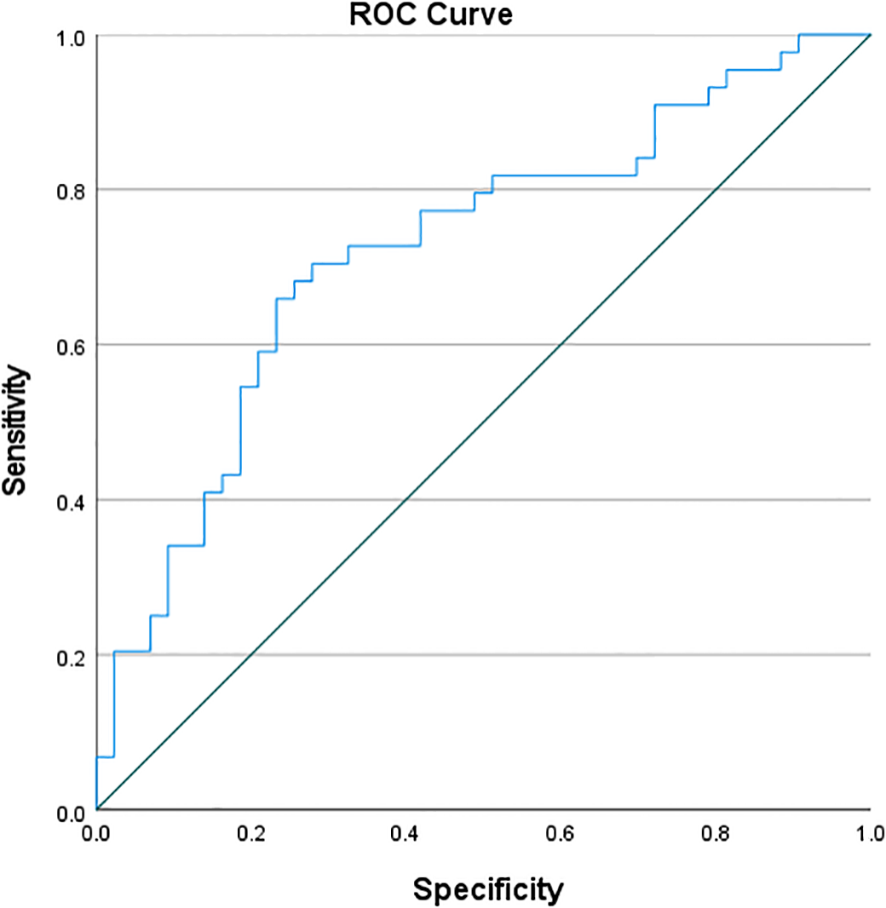
ROC curve of CD4^+^ lymphocyte counts for distinguishing PTB from NTMPD.

ROC curve analysis of CD4 showed an AUC of 0.722, with a sensitivity of 65.9% and specificity of 76.7%. The Youden’s index(**Table 6**; **Figure 1**).

**Table 6:** Differential diagnostic value of CD4 for the two diseases.

Variable	Sensitivity	Specificity	Youden’s index	P- value	AUC	95% CI (lower - upper)	
						Lower limit	Upper limit
CD4	0.659	0.767	0.426	0.000	0.722	0.614	0.830

AUC, Area Under the Curve.

## 4 Discussion

Pulmonary tuberculosis (PTB) and nontuberculous mycobacterial pulmonary disease (NTMPD), though both mycobacterial infections, differ significantly in etiology, treatment, and prognosis. Accurate differentiation between them is crucial. This study compared the clinical characteristics and peripheral blood lymphocyte subsets of PTB patients, NTMPD patients, and healthy controls to identify potential biomarkers for differential diagnosis.

In this study, the PTB and NTMPD groups showed no significant differences from the healthy control group in age and BMI, ensuring baseline comparability. Notably, the PTB group had a higher proportion of males and patients with diabetes mellitus compared to the NTMPD group. In contrast, the NTMPD group had a higher prevalence of structural lung disease, particularly bronchiectasis, and included relatively more female patients. These findings are consistent with previous studies (1, 2, 9, 10), reflecting the distinct host susceptibility factors for the two diseases. PTB tends to occur more frequently in males and diabetic patients, while NTMPD is closely associated with structural lung diseases, especially bronchiectasis. Moreover, the gender distribution of NTMPD differs in specific populations, such as elderly females with bronchiectasis.

The study found a significantly higher platelet count in the PTB group than in the NTMPD group. Platelets, key for hemostasis, also play an active role in inflammation and immune regulation (11). Active tuberculosis infection may boost platelet production via pro - inflammatory cytokines like IL - 6 and IL - 1β (12, 13). Activated platelets release mediators such as VEGF, IL - 1β, and PF4, potentially worsening pulmonary inflammation and tissue damage (13). While NTM infection can also activate platelets (14), proteomics studies indicate that this activation is less pronounced than in active tuberculosis (15). Our results suggest that an elevated platelet count could be a potential marker for differentiating PTB from NTMPD (16).Although platelet counts were statistically higher in PTB, the median difference was only 29.5 ×10^9^/L and overlaps with the normal reference range, limiting its clinical utility as a standalone discriminator.

Lymphocyte subset analysis was the study’s core finding. Lymphocytes play a key role in the anti - mycobacterial immune response (17, 18). The PTB group had significantly higher proportions of CD3^+^, CD4^+^, and CD45^+^ lymphocytes than the NTMPD group. CD4^+^ T cells are central to anti - mycobacterial immunity (19–21). They coordinate multiple effector mechanisms, such as activating macrophages (via IFN - γ), recruiting neutrophils, and regulating immune responses. Previous research has linked lower CD3^+^ and CD4^+^ lymphocyte proportions in PTB patients to higher bacterial loads and more extensive lesions (22). The higher CD4^+^ T cell proportion in the PTB group compared to the NTMPD group may be due to: 1.

Different infection characteristics: NTMPD is often a chronic, stealth infection. Long - term host - pathogen interaction may cause immune exhaustion or enhanced regulatory responses (23).2. Comorbidity effects: The high prevalence of structural lung diseases (e.g., bronchiectasis) in NTMPD patients can significantly impact local and systemic immunity (2, 24, 25), potentially suppressing or altering lymphocyte subset distribution. Third, functional assays (intracellular cytokine staining, activation markers) were not performed due to the retrospective design and lack of viable PBMCs. Future prospective studies should incorporate functional profiling to clarify whether the observed numerical differences translate into differential T-cell activity. Although samples were collected prior to specific therapy, we lacked standardized data on fever duration, radiographic extent, and acute-phase reactants at sampling. Residual confounding by disease severity cannot be excluded. Notably, In the multivariate model of **Table 5**, CD4 remained significant after simultaneous entry of CD3, CD45, and CD19 (OR = 0.992, 95% CI 0.984–1.000, P = 0.039).

Value of CD4^+^T Cells as a Diagnostic Marker: ROC curve analysis showed that the CD4^+^ T - cell proportion has good diagnostic efficacy in differentiating PTB from NTMPD (AUC = 0.722), with a sensitivity of 65.9% and specificity of 76.7%. This aligns with the findings of Xiao et al. (26), indicating that the CD4^+^ lymphocyte count is a potential peripheral blood indicator for distinguishing these two diseases. CD3^+^, a total T - cell marker, may mainly reflect changes in CD4^+^T cells. CD45, a common leukocyte antigen, is expressed on all nucleated blood cells, including lymphocytes, and is crucial for regulating T and B cell activation (27). Its upregulated expression may indicate a stronger immune activation state. In this study, the higher proportion of CD45^+^ lymphocytes in the PTB group likely reflects the stronger systemic immune activation induced by Mycobacterium tuberculosis infection (28).In future work we will construct a clinical-only prediction model (using platelet, neutrophil and lymphocyte counts) and quantify the incremental value of adding CD4^+^ lymphocyte counts beyond routine blood indices.

## 5 Summary and outlook

This study compared peripheral blood lymphocyte subsets between PTB and NTMPD patients, revealing significant differences in CD4^+^, CD3^+^, and CD45^+^ counts. The CD4^+^ T-cell count showed modest diagnostic performance (AUC = 0.722), suggesting it may serve as a supportive indicator once prospectively validated. These findings offer preliminary evidence for distinguishing PTB from NTMPD and could inform future treatment strategies. Although limited by its single-center design and sample size, this work provides a basis for larger, multicenter studies. We plan to validate the diagnostic utility of CD4^+^ and other subsets, develop optimized models, and explore their role in monitoring treatment response and prognosis across different mycobacterial infections.

## Data Availability

The raw data supporting the conclusions of this article will be made available by the authors, without undue reservation.
