# Supplementary material for: Comparative immunophenotyping of peripheral blood lymphocyte subsets in pulmonary tuberculosis and nontuberculous mycobacterial pulmonary disease: a retrospective study
Source: Front Immunol. 2025 Oct 15;16:1682099. doi: 10.3389/fimmu.2025.1682099 (PMC12568706; doi:10.3389/fimmu.2025.1682099)
Supplement: Supplementary file 1 [file SupplementaryFile1.docx]

Supplementary Table S1

Comparison of CD3, CD4, and CD45 Levels in PTB Patients with and without Diabetes

| Variable | Without diabetes (n = 59) | With diabetes (n = 19) | Statistical Measure | P- value |
| --- | --- | --- | --- | --- |
| CD3 | 831.43(494.39.-1071.31) | 731.26(552.42-937.39) | -0.339 | 0.735 |
| CD4 | 527.05(301.77-647.71) | 434.41(405.22-657.49) | -0.149 | 0.881 |
| CD45 | 1102.06(834.51-1514.07) | 1119.31(902.47-1433.87) | -0.068 | 0.946 |

Supplementary Figure S2. Distribution characteristics of CD4⁺ lymphocyte counts across groups.
The CD4⁺ counts in PTB, NTMPD and controls showed evident median differences (520, 331 and 990 cells/μL, respectively) and non-normal distributions; no extreme outliers (>3×IQR) were observed. Data are available upon request for readers who wish to generate their own box plots or violin plots.
